# A high-continuity and annotated tomato reference genome

**DOI:** 10.1186/s12864-021-08212-x

**Published:** 2021-12-15

**Authors:** Xiao Su, Baoan Wang, Xiaolin Geng, Yuefan Du, Qinqin Yang, Bin Liang, Ge Meng, Qiang Gao, Wencai Yang, Yingfang Zhu, Tao Lin

**Affiliations:** 1 State Key Laborary of Agrobiotechnology, Beijing Key Laboratory of Growth and Developmental Regulation for Protected Vegetable Crops, College of Horticulture, China Agricultural University, Beijing, China; 2 Genomics and Genetic Engineering Laboratory of Ornamental Plants, College of Agriculture and Biotechnology, Zhejiang University, Hangzhou, China; 3 State Key Laboratory of Crop Stress Adaptation and Improvement, School of Life Sciences, Henan University, Kaifeng, China

**Keywords:** *de novo* tomato genome, comparative genomics, high-density genetic map, QTL analysis

## Abstract

**Background:**

Genetic and functional genomics studies require a high-quality genome assembly. Tomato (*Solanum lycopersicum*), an important horticultural crop, is an ideal model species for the study of fruit development.

**Results:**

Here, we assembled an updated reference genome of *S. lycopersicum* cv. Heinz 1706 that was 799.09 Mb in length, containing 34,384 predicted protein-coding genes and 65.66% repetitive sequences. By comparing the genomes of *S. lycopersicum* and *S. pimpinellifolium* LA2093, we found a large number of genomic fragments probably associated with human selection, which may have had crucial roles in the domestication of tomato. We also used a recombinant inbred line (RIL) population to generate a high-density genetic map with high resolution and accuracy. Using these resources, we identified a number of candidate genes that were likely to be related to important agronomic traits in tomato.

**Conclusion:**

Our results offer opportunities for understanding the evolution of the tomato genome and will facilitate the study of genetic mechanisms in tomato biology.

**Supplementary Information:**

The online version contains supplementary material available at 10.1186/s12864-021-08212-x.

## Background

Tomato (*Solanum lycopersicum*) is an important model plant for scientific researches on fruit development and quality [1]. The tomato cultivation area has increased by ~1 million hectares over the past decade, and the yield has increased from 155 million tons to 181 million tons (http://www.fao.org). As a nutritious vegetable that contributes to the human diet, tomato is reported to contain more health-promoting compounds such as lycopene than some other popular fruits. These compounds lower risk of cancer and maintain human health [2]. Tomato was originally found mainly in the Andean mountains of South America. Its fruit weight and quality differ markedly among different horticultural groups, and wild tomatoes have smaller seeds and lower yields than cultivars.

A draft genome of the tomato cultivar Heinz 1706 produced using shotgun sequencing technology was released in 2012 [3] and widely used as a reference genome for scientific researches. However, the fragmented nature of this genome and the resulting incomplete gene models could hindered the discovery and functional analysis of important genes. The completeness, accuracy, and contiguity of genome assemblies depend mainly on sequencing technology and assembly strategy. In the current genomic era, single-molecule real-time (SMRT) sequencing technology and new assembly pipelines have remarkably improved the quality of genome assemblies such as those of rice [4], cucumber [5], and tomato [6]. Although these genome assemblies have accelerated some scientific researches, such as QTL mapping and transcriptome analysis, it is far from sufficient for understanding more comprehensive and personal precision breeding. After several updates of tomato genome, the SL4.0 tomato genome has reached chromosomal assembly, but its continuity and integrity remains to be further improved to facilitate the identification of large structural variations and gene mining.

Across over the past ~10,000 years, humans have selected consciously and unconsciously for beneficial traits that have made wild plants more suitable for human use. The impacts of human selection on crops have been recorded in their genomes and have a central role in crop improvement. The resulting “domestication syndrome” comprises a common suite of traits useful for human needs and plant survival, such as larger fruits or grains, better taste, bigger seeds, and more robust plants overall [7, 8]. In crop species, bigger seeds can accumulate adequate nutrition for germination and produce more vigorous seedlings, and effectively increase yield and utilization in grain crops [9]. Seed size, as a domestication trait, has been elucidated in several crops, and genes such as *GS3* [10, 11], *DEP1* [12], *GW2* [13], *GW8* [14], and *qSW5* [15] in rice; *HvYrg* in barley [16], and *TaGW2* in wheat [17] have been characterized. But small seeds could affect the fruit size and quality in orange [18] and watermelon [19]. Therefore, it is crucial to explore the balance between seed size and other traits in crops. In tomato, seeds are smaller in wild tomatoes than in cultivars. *Sw4.1* is one of the major QTLs which encode an ABC transporter responsible for phenotypic variation in tomato seed size [20, 21].

In this study, we generated a highly continuous and complete genome sequence of Heinz 1706 (version SLT1.0) that contains many fewer gaps and unplaced contigs and demonstrates better assembly of repetitive regions. By comparing the genomes of *S. lycopersicum* and *S. pimpinellifolium* LA2093, we found a large number of genomic fragments that appear to be likely involved in domestication. We also used a RIL population, which was derived from a cross between the cultivated line OH88119 and the wild line PI128216, to identify candidate genes and loci that control several important agronomic traits. Our work offers new opportunities for understanding the evolutionary history of the tomato genome and the genetic mechanisms that underlie complex traits in tomato breeding.

## Results

### High-quality genome assembly

We assembled a highly continuous and complete genome sequence of Heinz 1706 using an integrated genome sequencing approach that combined 131.78 Gb (168.52×) of SMRT data, 226.97 Gb (290.24×) of BioNano data, 140.52 Gb (179.70×) of Hi-C data, and 50.93 Gb (61.53×) of Illumina short-read data (Supplementary Table S1). The PacBio long reads with an N50 read length of 32.82 kb were assembled with CANU software [22], generating a 875.21-Mb genome with a contig N50 of 17.83 Mb (Table 1). To reduce fragmentation and order the contigs, BioNano data and Hi-C data were used to assist with scaffold construction using RefAligner and Assembler [23], HERA [24], and Juicer [25] software. A Hi-C-based physical heatmap comprising 12 groups was generated (Supplementary Fig. S1) and used to create 12 pseudo-chromosomes that anchor ~790.59 Mb of the genome and harbor 97.61% (33,562) of the predicted protein-coding genes. The genome assembly was polished with Illumina short reads for error homozygous SNPs or indels using Pilon software [26]. As a result, we generated a 799.09-Mb genome assembly, namely SLT1.0.

**Table 1:** Genome assembly and annotation of SLT1.0

	SLT1.0	SL4.0	SL3.0
Genome assembly (Mb)	799.09	782.52	828.08
Non-N bases	797,955,212	782,475,302	746,357,470
Number of gaps	210	286	22,700
Number of total contigs	1,615	504	-
Longest contig length (Mb)	47.16	26.29	-
N50 of contigs (Mb)	17.83	6.01	-
Number of unplaced contigs	112	176	4,374
Unplaced contigs sequence length (Mb)	8.50	9.64	20.85
Number of genes	34,384	34,075	35,768
Percentage of gene length in genome (%)	16.21	15.56	17.33
Mean gene length (bp)	3,766.53	3,572.44	4,011.09
Gene density (per Mb)	43.03	43.55	43.19
Mean coding sequence length (bp)	223.02	228.01	219.97
Mean exon length (bp)	310.11	275.03	308.36
Mean intron length (bp)	270.41	606.69	632.38
Masked repeat sequence length (Mb)	558.49	546.95	507.14
Repeats percentage of genome size (%)	69.89	69.90	61.24

The conserved genes from the Benchmarking Universal Single-Copy Orthologs (BUSCO) gene set [27] were used to gauge the accuracy and completeness of the SLT1.0 assembly. The results showed that the SLT1.0 assembly contained 97.70% complete genes and 0.30% fragmented genes. The value of the LTR Assembly Index (LAI) was 12.41, which was consistent with that of the previously released SL4.0 tomato reference genome (LAI 12.54). Whereas, the SLT1.0 genome had only 210 gaps, which is less than 286 and 22,700 in the SL4.0 and SL3.0 genomes, respectively (Table 1). More than 99.88% of the genome assembly had greater than one-fold coverage with Illumina short reads. All these evidences demonstrated the high continuity and completeness of the SLT1.0 genome assembly.

### High-quality genome annotation

**Fig. 1:**
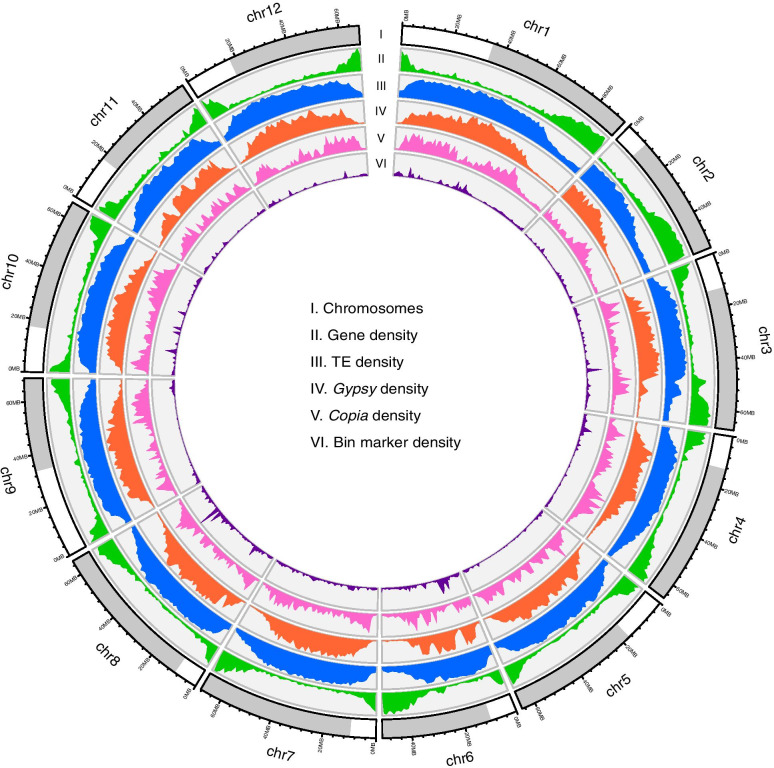
Genomic landscape and structural variants of *S. lycopersicum* cv. Heinz 1706. (i) Ideogram of the 12 chromosomes with scale in Mb, with centromere positions marked between gray and white. (ii) Gene density along each chromosome (number of genes per Mb). (iii) Repeat content along each chromosome (% nucleotides per Mb). (iv) *Gypsy* retrotransposons content (% nucleotides per Mb). (v) *Copia* retrotransposons content (% nucleotides per Mb). (vi) Bin marker content (% nucleotides per Mb)

**Fig. 2:**
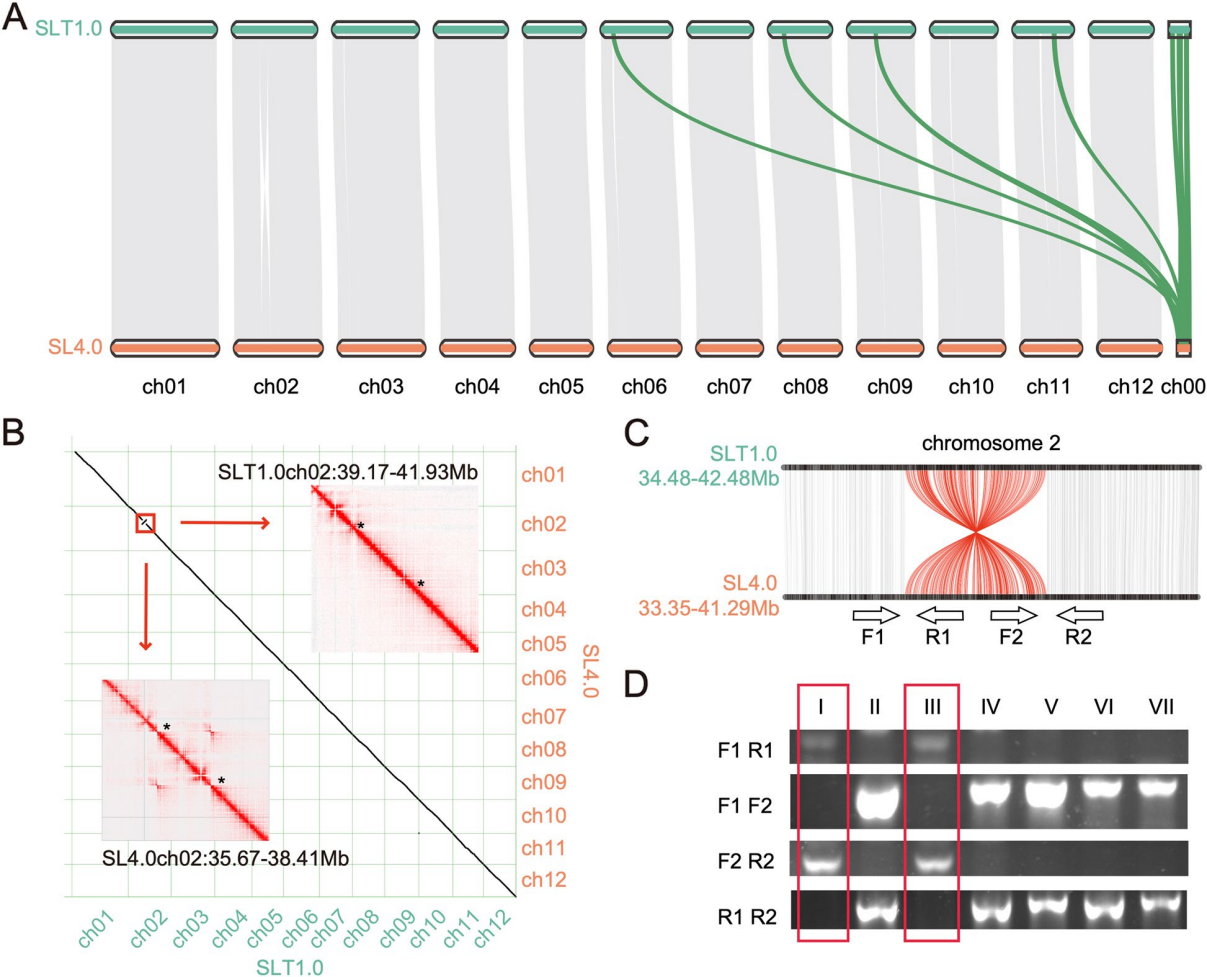
Alignment between the Heinz 1706 SLT1.0 and SL4.0 genomes. **A** Genome collinearity analysis showed that four scaffolds from SL4.0 are placed on chromosomes of the SLT1.0 genome and that there is an inversion on chromosome 2. **B** The color intensity of the Hi-C heatmap represents the number of links between two 25-kb windows. The presence of an inversion is supported by high-density contacts indicated by two asterisks in the Hi-C heatmap generated from SL4.0 Hi-C reads (lower left), whereas no corresponding contact is found in the SLT1.0 Hi-C heatmap (upper right). **C** The inversion shown in red on chromosome 2. F1, R1, F2, and R2 are primers around the break points. The blots are cropped. **D** Seven Heinz 1706 individuals were identified, two of which (I, III) had inversions

Except for *ab initio* prediction and protein-homology-based prediction, we also used transcriptome data, including the bulked RNA-seq data with a mapping rate of 99.73%, and previously-released RNA-seq data from various tissues [3] with a mapping rate of 97.97%, to facilitate gene annotation of the assembled genome. In total, we predicted 34,384 protein-coding genes with an average length of 3,766.53 bp and 6.55 exons per gene in the SLT1.0 genome (Table 1 and Supplementary Table S2). Gene completeness was estimated to be 98.20% based on the BUSCO gene set, higher than that (92.9%) in the SL4.0 genome [27], and the protein-coding genes were unevenly distributed along the chromosomes (Fig. 1). Comparative analysis showed that each gene in a gene set (in total 234 genes) in the SLT1.0 genome corresponded to more than two genes in the SL4.0 genome (Supplementary Table S3). Gene collinearity analysis identified 33 collinear gene blocks between the SLT1.0 and SL4.0 genomes, harboring 28,892 (84.03%) and 28,389 (83.30%) homologous genes, respectively (Fig. 2A, Supplementary Fig. S2). Some unplaced contigs in the SL4.0 genome were successfully assigned to chromosomes 6, 8, 9 and 11 in the SLT1.0 genome, which contained 100 genes in the SLT1.0 genome. These results highlight the high accuracy and completeness of the SLT1.0 genome assembly and gene models.

A comprehensive analysis of the genome sequences identified 965 collinear chromosomal blocks between the SLT1.0 and SL4.0 genomes. These blocks contained 32,922 and 32,554 genes, accounting for 95.75% and 95.54% of the SLT1.0 and SL4.0 genomes, respectively. Genome alignment revealed that 97.52% of the SLT1.0 genome accounted for 99.58% of the SL4.0 genome, including 27,417 SNPs and 119,246 Indels. However, we detected a 2.76 Mb inversion from 39.17 to 41.93 Mb on chromosome 2 of the SLT1.0 genome (Fig. 2B). The continuous interaction signals on the Hi-C heatmap, as well as PCR and Sanger sequencing, showed that this region was not misassembled (Fig. 2B-C, Supplementary Fig. S3, Supplementary Table S4). This result indicated that heterozygous variation may exist in the previously reported Heinz 1706 accession.

### Transposable element analysis

**Fig. 3:**
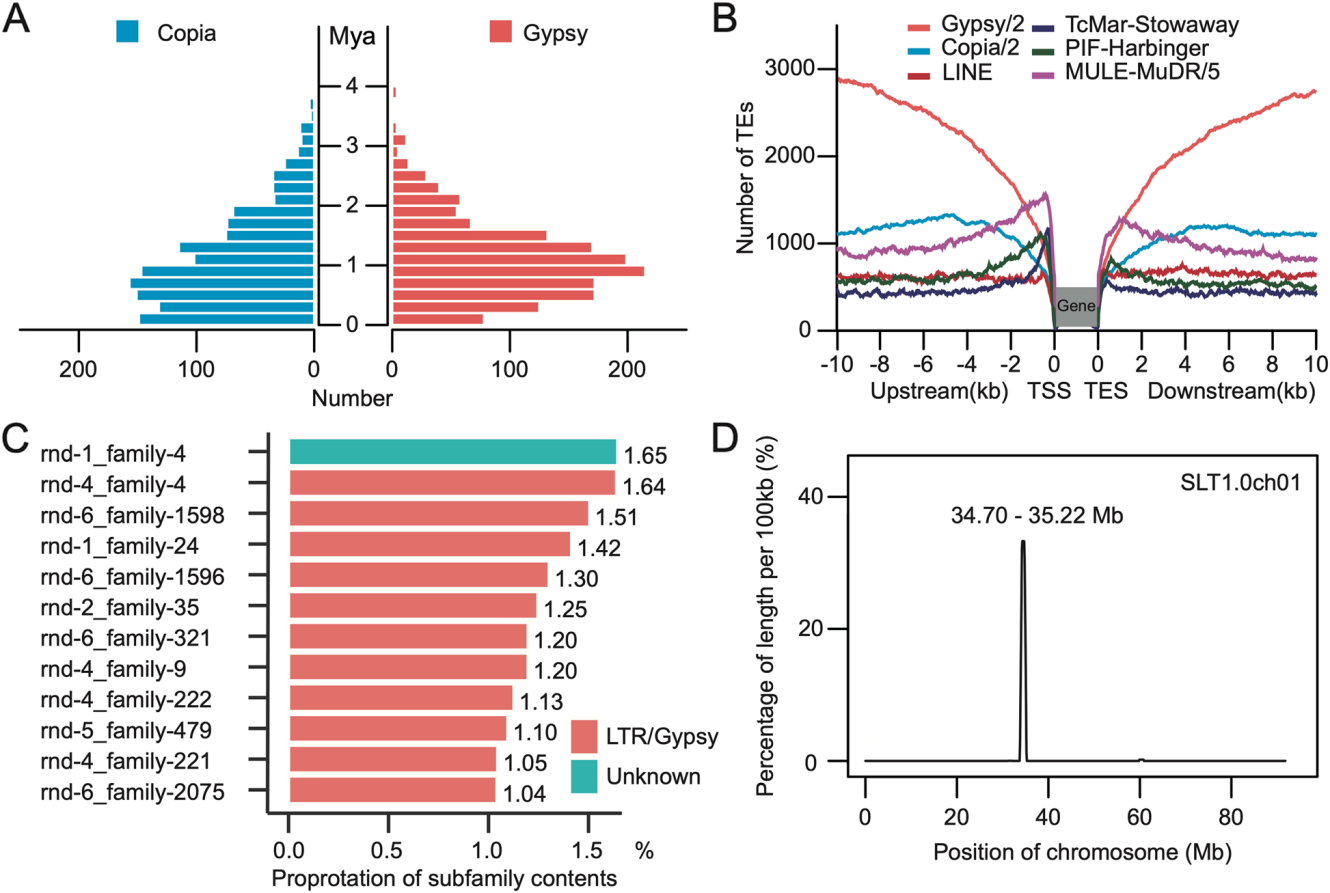
Repetitive sequence analysis. **A** The estimated insertion time of LTR retrotransposons, showing the statistics of *Gypsy*-type LTRs in red and *Copia*-type LTRs in blue. **B** Frequencies of transposable elements (TE) in the vicinity of genes. *Gypsy* and *Copia* had the highest frequencies in the intergenic region and the lowest frequencies near the gene regions. *LINE*, *TcMar-Stowaway*, *PIF-Harbinger*, and *MULE-MuDR* exhibited the opposite distribution pattern. **C** The top 12 TE subfamilies with the longest length, including 11 *Gypsy* and one *Unknown*-type subfamily. **D** The frequency distribution of the *Unknown*-type rnd-1_family-4 subfamily, showing that it was enriched towards the centromere of chromosome 1

A total of 524.84 Mb of repetitive sequences were identified, accounting for 65.66% of the SLT1.0 genome assembly, which was similar to that reported in the SL4.0 genome (508.89 Mb, 65.03%) (Supplementary Table S5). Among these repetitive sequences, long terminal repeats (LTRs) were the predominant TE family, covering 50.25% (401.60 Mb) of the genome. *Gypsy*-type LTRs (344.52 Mb) were the most common subfamily and six times more abundant than *Copia*-type LTRs (57.09 Mb), slightly higher than the *Gypsy*-type LTRs (342.44 Mb) and *Copia*-type (56.60 Mb) in the SL4.0 genome. We used a combination of methods, including LTR-FINDER [28], LTR-Harvest [29], and LTR-Retriever [30], to identify intact LTRs. A total of 3,220 LTRs were detected in the SLT1.0 genome assembly, including 1,553 *Gypsy*-type LTRs and 1,346 *Copia*-type LTRs. The estimated insertion time of the LTR retrotransposons showed that *Gypsy* and *Copia*-type LTRs had a recent and similar burst 0.60-1.00 million years ago (Mya) (Fig. 3A), and were enriched far from coding genes (Fig. 3B). These results indicated that the burst of *Gypsy*-type LTRs may be the major driving force for the expansion of the tomato genome.

To identify the centromere regions, we detected the top 12 TE subfamilies, including 11 *Gypsy* and one unknown-type subfamilies, which together comprised over 15.47% of the genome (Fig. 3C). The density of these TE subfamilies along all the chromosomes showed that only the *Unknown*-type rnd-1_family-4 subfamily (1.65% of the genome) was enriched near centromeres but absent from the rest of the genome (Fig. 3D, Supplementary Fig. S4). In addition, we found that 65.21% of the unanchored Contig/Scaffold sequence length comprised highly repetitive regions. Overall, we predicted 12 potential centromeric regions ranging from 1.90 to 6.90 Mb on the 12 chromosomes.

### Comparison of the SLT1.0 and *S. pimpinellifolium* LA2093 genomes

**Fig. 4:**
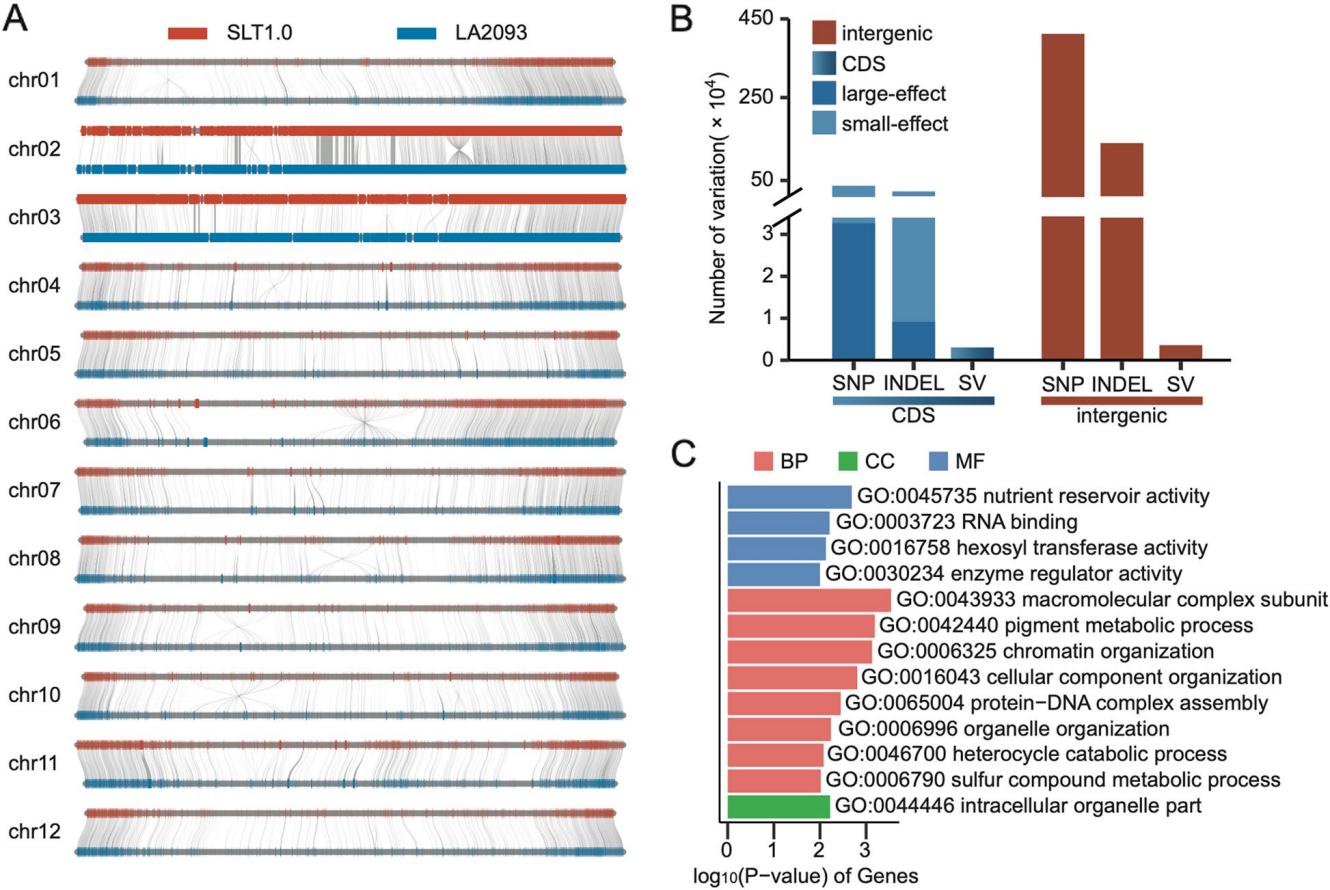
Alignment between the SLT1.0 and *S. pimpinellifolium* LA2093 genomes. **A** Gene colinearity of the SLT1.0 and LA2093 genomes. The red bar represents the SLT1.0 chromosome, the blue bar represents the LA2093 chromosome, and the gray lines represent the collinear regions. **B** Numbers of SNPs, indels, and structural variation in CDS and intergenic regions. The numbers of SNPs with nonsynonymous mutations (large-effect), SNPs with synonymous mutations (small-effect), and SNPs in intergenic regions, as well as the number of non-triple (large-effect) indels, triple (small-effect) indels, and indels in intergenic regions were further shown. **C** GO terms enriched in genes affected by SNPs and indels selected during domestication

Structural variations (SVs) between wild and cultivated species can cause many phenotypic differences in domestication traits such as fruit weight and quality [31]. Based on protein homologies between the SLT1.0 and LA2093 genomes, we found that 23,544 genes (68.47%) in the SLT1.0 genome had one-to-one collinear relationships with 23,474 genes (65.64%) in the LA2093 genome (Fig. 4A). In addition, genome collinearity analysis showed that syntenic genomic blocks occupied 95.63% of the SLT1.0 genome and 96.67% of the LA2093 genome, respectively. We also identified 6,647 SVs (more than 1 kb in length) between the SLT1.0 and LA2093 genomes, including 3,054 (45.95%) SVs in 2,862 genes (Fig. 4B). GO analysis showed that these genes were significantly enriched in the function of oxidation-reduction process, photosynthetic electron transport chain and proton-transporting ATP synthase complex (Supplementary Fig. S5). We also identified 4,493,889 SNPs and 2,459,597 indels between the two genomes (Fig. 4B), including 418,844 SNPs and 245,310 indels located in 29,862 genes. We noted that 45,229 nonsynonymous SNPs resided in 18,178 genes and 9,148 frameshift indels in 1,559 genes, including 7,788 located in domestication regions [32]. They were significantly enriched in macromolecular complex, pigment metabolic process, nutrient reservoir activity, and intracellular organelle parts (Fig. 4C), suggesting these genes may have contributed to disease resistance and fruit traits during tomato domestication.

### High-density genetic map construction

**Fig. 5:**
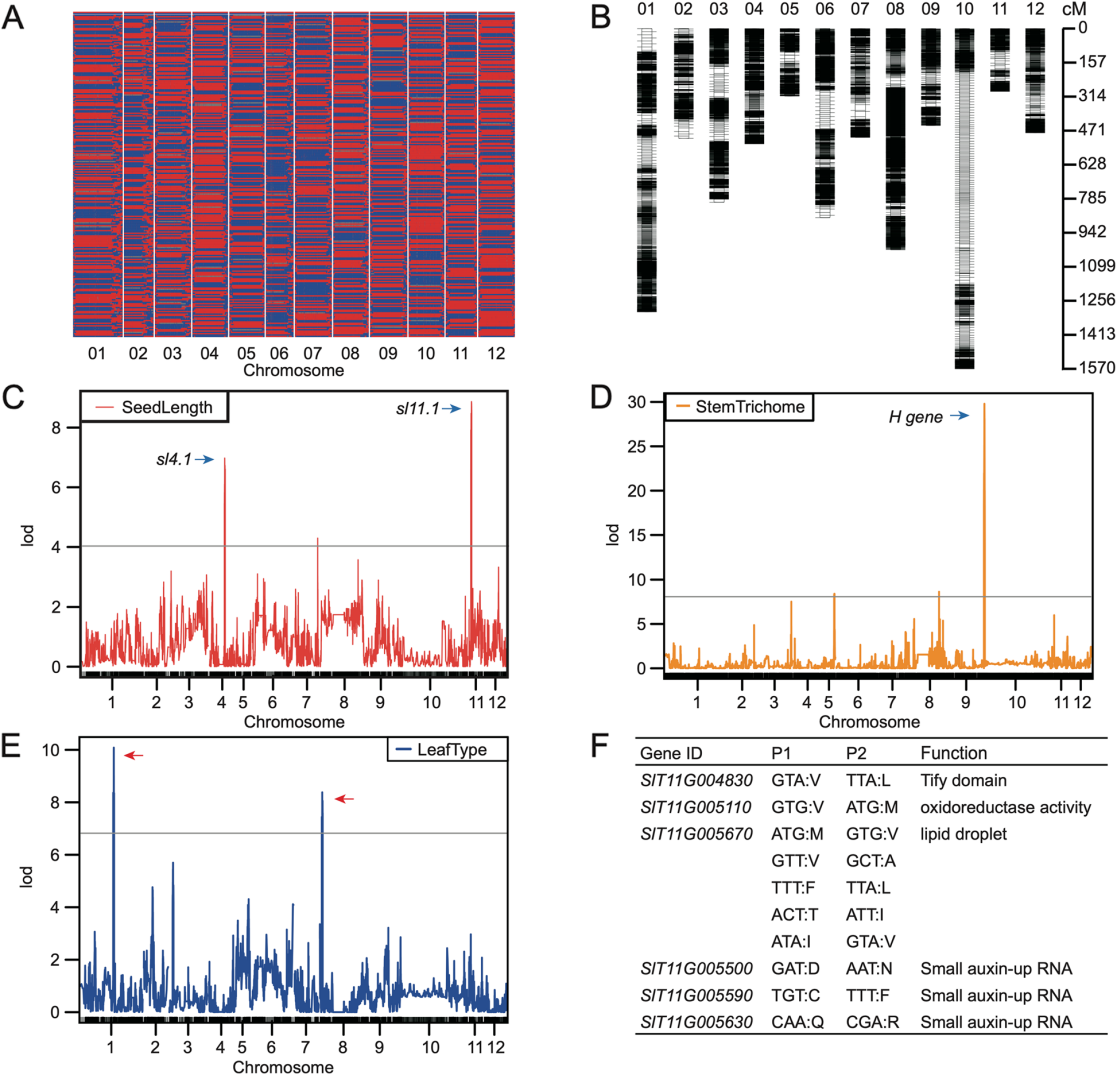
Genetic map construction and QTL mapping of the RIL population. **A** Graphical representation of the re-sequencing-based mapping results of 247 RILs sorted according to the SLT1.0 genomic location. **B** High-density genetic map constructed by RIL population. **C** Mapping of QTLs across the entire RIL population for seed length, **D** stem trichomes density **E** and leaf type traits. **F** Seed-size-related candidate genes and non-synonymous mutations between the cultivated line OH88119 and the wild line PI128216.

A high-quality tomato reference genome can provide new insights into the genetic basis of important agronomic traits. Here, we constructed a RIL7 population of 247 progenies derived from a cross between the cultivated line OH88119 (64.27×) and the wild line PI128216 (42.35×). Resequencing of these progenies generated 1.7 Tb of data with an average depth of 6.91× and 97.98% coverage of the SLT1.0 genome. After aligning the reads to the genome, we identified 4,739,716 SNPs between the parental lines, 2,818,901 (59.35%) of which were genotyped in the RIL7 population. To construct a high-density tomato genetic map, we employed a Hidden Markov Model (HMM) to infer all recombination events in the RIL population. A total of 17,726 recombination events were detected across the whole genome, with one recombination event at an average interval of 3.78 kb (Fig. 5A). We found that 1,477 crossovers per chromosome ranged from 1.01 kb to 7.92 Mb in size and that the recombination rate varied across the genome with an average of 11.22 cM/Mb (Fig. 5B, Supplementary Fig. S6). Furthermore, recombination rate increased with distance from the centromeric regions on chromosomes 5 and 9, but this trend was not evident on the other chromosomes (Fig. 1).

We defined 35,836 raw bins (an average physical length of 22.06 kb) across the entire RIL population and constructed a high-quality set of 17,741 bin markers with a physical length of greater than 1 kb and a minor allele frequency of greater than 0.05. Based on recombination rate, we created a genetic linkage map of 13,973 unique bin markers anchoring twelve linkage groups that corresponded to the twelve chromosomes (Fig. 5B). This genetic map spanned 786.81 Mb, representing more than 98.46% of the assembled SLT1.0 reference genome. Taken together, these analyses produced a high-quality genetic map for genetic research on important agronomic traits in tomato.

### QTL mapping of important agronomic traits in tomato

The RIL population exhibited diversity for several important agronomic traits. The cultivated line OH88119 has a larger fruit and seed size, both of which are key traits associated with human selection in tomato. To gauge the accuracy of the genetic bin map, we performed an association study on three agronomic traits (seed size, leaf architecture, and trichomes) and identified five significantly associated QTLs in the SLT1.0 genome (Fig. 5C-E). Plant glandular trichomes can produce secondary metabolites that defend against herbivores and pathogens [33, 34]. Previous studies have shown that a 618-bp *H* gene encoding a C2H2 zinc finger protein regulates the formation of multicellular trichomes in tomato leaves [35]. Here, the leaf trichome locus was localized to a smaller interval of 2.82 kb that included two bins on chromosome 10 (Fig. 5D), suggesting the high quality of the genetic bin map.

The seed is an important plant reproductive organ, but larger seeds affect fruit taste, reducing the economic value of the berry crop. We used the genetic bin map to identify two seed size QTLs above the LOD threshold associated with seed length (Fig. 5C). These major-effect QTLs were resolved into a 1.32-Mb region with 40 bins ranging from 5.75 to 7.07 Mb on chromosome 4 (*sl4.1*) and a 0.74-Mb region with 36 bins ranging from 4.08 to 4.82 on chromosome 11 (*sl11.1*). Intriguingly, we found that *sl11.1* on chromosome 11 was located in domestication sweep DS169 [32], indicating that it may have played an essential role during human selection. We identified 11,709 parental SNPs in this region, including 303 nonsynonymous variations in 87 genes. Among these SNPs, 132 nonsynonymous variations in 65 genes were also present in the RIL population. After exploring their functions, we identified six candidate genes for seed length, including a TIFY domain protein, a lipid droplet-associated protein (LDAP)-interacting protein (*LDIP*), and three small auxin-up RNA (SAUR) proteins (Fig. 5F). In particular, one gene encoding an *LDIP*, *SlT11G005670*, appeared to be a strong candidate for seed length. Based on homology comparison, we found that the *LDIP* gene influences lipid droplet size and neutral lipid homeostasis in *Arabidopsis* seeds [36].

## Discussion

A highly contiguous and complete genome assembly is a powerful tool for molecular genetic studies of agronomic traits in tomato. Since the tomato cultivar Heinz 1706 is considered as a reference genome for scientific researches [3], its precise and complete genome has be updated using the advanced assembly and annotation technology. However, the continuity of genome assembly is still a challenge to the tomato genome. In this study, we combined PacBio, BioNano, and Hi-C data to produce the high-quality and contiguous SLT1.0 tomato genome. The 799.09-Mb assembly had an N50 of 17.83 Mb, and more than 98.94% of its sequences were anchored to 12 chromosomes. The SLT1.0 genome had more repeats sorted and anchored to chromosomes than the previously released SL4.0 genome. In the SLT1.0 genome, the number and length of LTRs, mainly *Gypsy*-type LTRs and *Copia*-type LTRs, were longer than the SL4.0 genome, indicating a more accurate assembly of the SLT1.0 genome. Analysis of repeat subfamilies showed that a specific subfamily, rnd-1_family-4, was found in centromeric regions of the SLT1.0 genome, whereas a similar reliable repeat family was not detected in the SL4.0 genome [6]. Therefore, we could determine the centromere regions in the SLT1.0 genome. Comparative genome analysis revealed that a 2.76-Mb inversion was present on chromosome 2 in SLT1.0 relative to SL4.0 (Fig. 2). The inversion was validated by Sanger sequencing and contained no functional genes in adjacent breakpoints, suggesting it is a continuous fragment that has no effect on the SLT1.0 genome. However, we must be cautious and further verify these different fragments between the SLT1.0 and SL4.0 genomes.

Because of the small size and poor taste of wild species, fruit mass and quality are important domestication traits in tomato. Human domestication and improvement have increased the size of modern tomato fruits about 100 times relative to their ancestors [32]. At the same time, the flavor and resistance of tomatoes have been greatly reduced [37]. Comparative genomic analysis of the SLT1.0 and *S. pimpinellifolium* LA2093 genomes found that genes located in the domesticated regions had large effects including non-synonymous and frameshift mutations. These genes are mainly involved in the macromolecular complex and pigment metabolic process, which could be possibly selected by humans for a long time. Compared with SNPs and small indels, structural variations had high levels of heritability in plants [38]. We identified structural variations in some important genes that affect tomato fruit weight, such as *SlT11G021690* (oxidoreductase activity). This result suggests that structural variations in breeds were more likely to have been fixed in cultivars during the process of human selection. Furthermore, these structural variations may be used as potential targets for future breeding programs to improve fruit mass and quality.

In the process of crop domestication, human beings have paid particular attention to yield, shelf-life, and resistance to biotic stresses [7, 39], especially seed and fruit development, but human have different perference for different species. It seems that people are inclined to select larger seeds to improve the emergence rate, yield, quality, and other important traits, especially in edible seed crops such as rapeseed [40] and bean [41]. However, size and number of seed affect the taste and reduce the quality of edible fruits. Meanwhile, small seeds will affect the fruit flavor and germination rate, the balance between seed size and fruit flavor is particularly important in some species such as orange [18] and watermelon [19]. Here, QTL analysis of 247 tomato RIL populations indicated that the *SlT11G005670* gene may have influenced tomato seed length during domestication. By extracting the SNP sites of the parents, we found that the gene contained a nonsynonymous mutation in the cultivated tomato line. We also found that *LDIP* genes from other species have high homology with *SlT11G005670* affecting lipid droplet size and neutral lipid homeostasis in *Arabidopsis thaliana* [36]. These findings have potential contributions to future tomato breeding and fruit quality improvement. However, more evidence are required to understand the potential molecular mechanisms by which this gene controls seed length.

## Conclusion

Overall, we produced a high-quality tomato genome that will facilitate the molecular dissection of important agronomic traits in tomato. We generated a high-density genetic map and detected five QTLs related to seed and leaf traits. We also identified six candidate genes in two genomic regions that appear to control differences in seed length. This high-quality genome and high-density genetic map will be powerful tools for tomato breeding and can deepen our understanding of tomato biology.

## Methods

### Plant materials and sequencing

Plants were grown in the greenhouse in China Agricultural University in Beijing, with a 16 h light/ 8 h dark cycle. The cultivated line OH88119 was crossed with the wild line PI128216 to create the F_1_ progeny, and RIL population was developed through single-seed-descent. Fresh leaf tissues were collected from each line of RIL population and resequenced on the Illumina NovaSeq 6000 platform. A PacBio SMRT library of *Solanum lycopersicum* accession Heinz 1706 was constructed and sequenced on the PacBio Sequel II platform. The Hi-C libraries were prepared following the Proximo Hi-C plant protocol with HindIII as the restriction enzyme for chromatin digestion. The Hi-C libraries were sequenced on the Illumina NovaSeq platform with a read length of 150 bp. For optical mapping, high-molecular-weight DNA was isolated and labeled using a Bionano Saphyr System. The RNA of mixed tissues including root, stem, leaf, flower, and fruit, were extracted using Promega RNG extraction kit (Promega Biotech, Beijing, China), and sequenced.

### De novo genome assembly

The raw SLT1.0 SMRT reads were corrected and assembled into sequence contigs using CANU with default parameters. The contigs were used for HERA assembly with the corrected SMRT reads. To identify sequence overlaps, all contigs and corrected reads were aligned all-against-all using Minimap2 [42] and BWA [43] with default parameters. The HERA-assembled super-contigs were combined with BioNano genome maps to generate hybrid maps using IrysView software (BioNano Genomics) with a minimum length of 150 kb. The resulting contigs were further clustered basing on the Hi-C data using 3D-DNA software [44] with the default parameters. Pilon [26] was used for further error correction with three rounds of polishing.

### Repeat analysis and gene annotation

The integrity of the final genome assembly was assessed in conjunction with BUSCO (v4.1.4) [27] using Benchmarking Universal Single-Copy Orthologs. A combination of *de novo* and homology-based methods was used to identify interspersed transposable elements (TEs). A *de novo* repeat library was built using RepeatModeler (v2.0.1) [45] and LTR_retriever (v2.9.0), [30]. Both the *de novo* library and RepBaseRepeatMaskerEdition-20181026, which is the most commonly used repetitive DNA element database, were used to identify TEs with RepeatMasker (v4.1.0) [46].

The RNA-Seq reads from this study were used to predict protein-coding genes in the repeat-masked SLT1.0 genome [3]. The cleaned high-quality RNA-Seq reads were aligned to the assembled genome using HISAT2 [47] with default parameters, and the read alignments were assembled into transcripts using StringTie [48]. The complete coding sequences (CDS) were predicted from the assembled transcripts by the PASA pipeline [49]. The BRAKER [50], GeneMark-ET [51], and SNAP [52] softwares were performed on *ab initio* gene predictions. Finally, high-confidence gene models were predicted by integrating *ab initio* predictions, transcript mapping, and protein homology evidence with the MAKER pipeline [53].

### Genome comparisons and SV identification

Genome comparisons between SLT1.0 and SL4.0 and between SLT1.0 and LA2093 were performed via whole-genome alignment using the MUMmer package (v3.23) [54]. The one-to-one alignment blocks were identified using delta-filter program. Then the show-snp tools were used to identify SNPs and indels using uniquely aligned fragments, and the show-diff tool statistics were used to screen for structural variations over 1 kb in length. The SnpEff [55] software was used to analyze the various SNPs and indel types on the chromosomes.

### Genetic map construction and QTL analysis

We identified SNPs across the 247 F_7_ RILs and the two parents using BWA (v0.7.10) [43] and samtools (v0.1.19) [56] softwares. The high-quality SNPs were called by bcftools (v1.10.2) [57], and SNPs were further filtered to retain only those with different homozygous genotypes in both parents, of which the quality ≥ 30, MQ ≥ 30, 2 ≤ AF1 ≤200. We generated a genotype matrix from the 247 RILs , and genetic distances were calculated using MSTMap [58]. The resulting bin marker data were imported into MG2C (v2.0) (http://mg2c.iask.in/mg2c_v2.0) to construct the genetic map.

QTL analysis was performed using trichomes, leaf type, and seed size phenotype data from 247 RIL population samples. For the trichomes phenotype, we divided it into five grades based on its density and length, and tomato leaf types are classified into four types according to the shapes. These two phenotypes in RIL population were independently estimated by three persons and the final phenotypes were taken from the average of them. To investigate seed size traits, we measured seed length with three biological replicates. The maximum likelihood estimation method was used to calculate the recombination rate and LOD values between bin markers. The bin markers with LOD value greater than 3.0 were selected as QTLs. The candidate genes were identified based on non-synonymous SNP mutations in both parents and their homologs in other species. We determined the function of these gene based on the homologs genes of the NCBI database (https://www.ncbi.nlm.nih.gov/).

## Supplementary Information

**Additional file 1.**

**Additional file 2.**

**Additional file 3.**

**Additional file 4.**

**Additional file 5.**

**Additional file 6.**

**Additional file 7.**

**Additional file 8.**

**Additional file 9.**

**Additional file 10.**

**Additional file 11.**

## Data Availability

All the raw sequencing data for genome assembly and annotation have been deposited into the Genome Sequence Archive (GSA) database (http://bigd.big. ac.cn/gsa) in BIG Data Center under Accession Number PRJCA004585. Information for the assembled genome SLT1.0 was deposited both into the Genome Warehouse (GWH) database (https://bigd.big.ac.cn/gwh/) in the BIG Data Center under Accession Number GWHBAUD00000000, and the NCBI database under Accession Number PRJNA757583.
