# Supplementary figures and images for: A high-continuity and annotated tomato reference genome
Source: BMC Genomics. 2021 Dec 15;22:898. doi: 10.1186/s12864-021-08212-x (PMC8672587; doi:10.1186/s12864-021-08212-x)

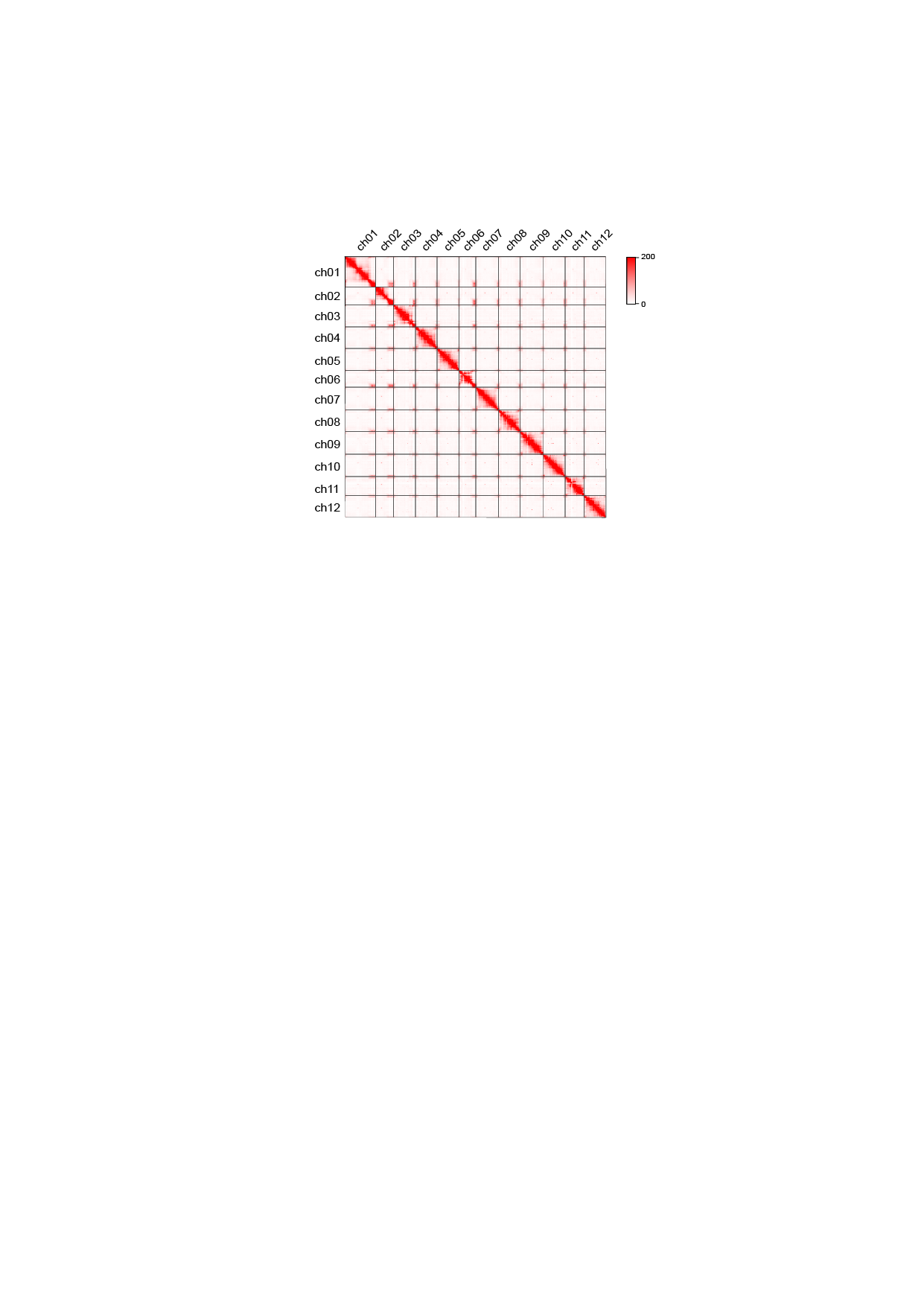

Supplement: Supplementary file 2 — Additional file 2. [file 12864_2021_8212_MOESM2_ESM.png]

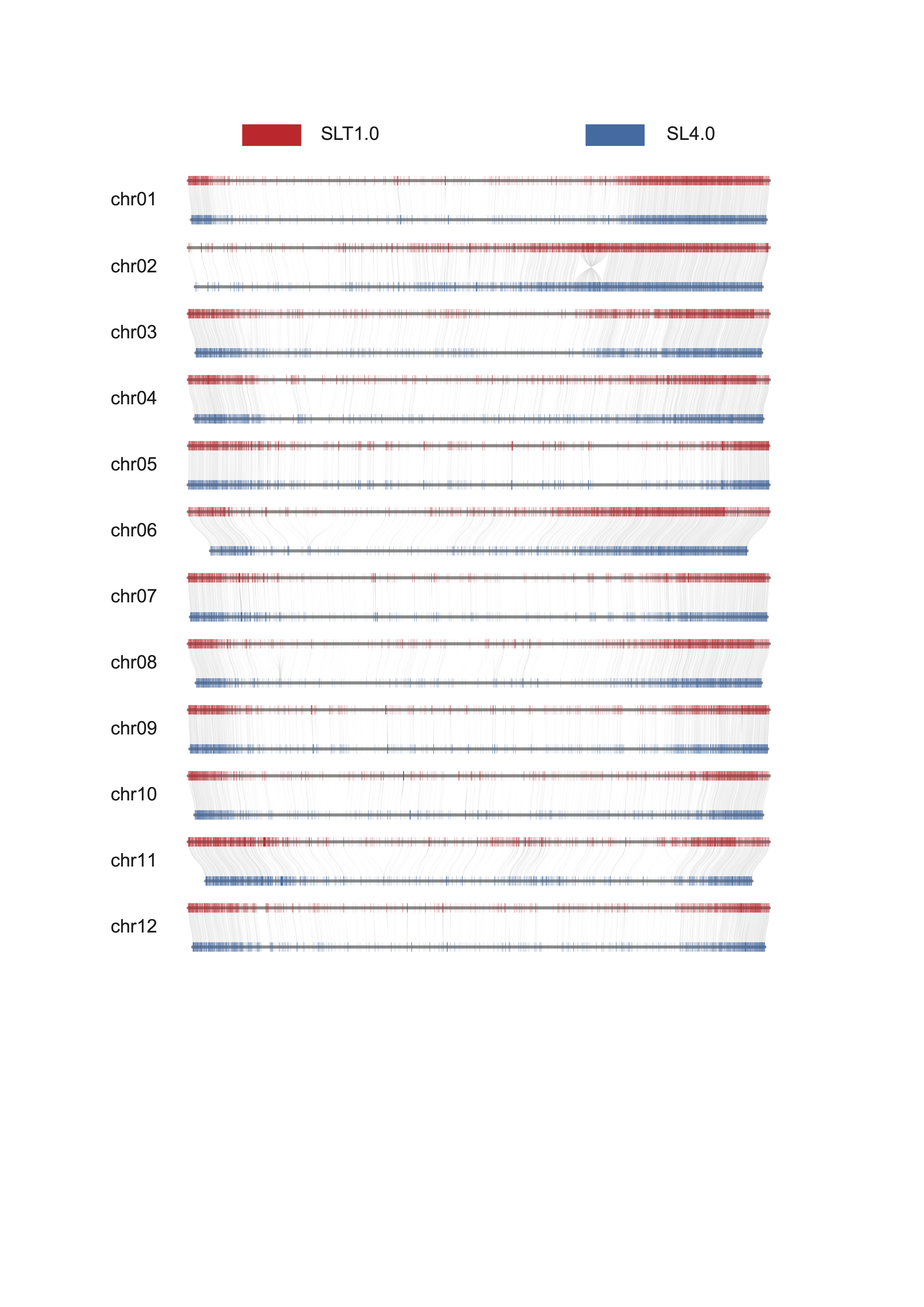

Supplement: Supplementary file 5 — Additional file 5. [file 12864_2021_8212_MOESM5_ESM.png]

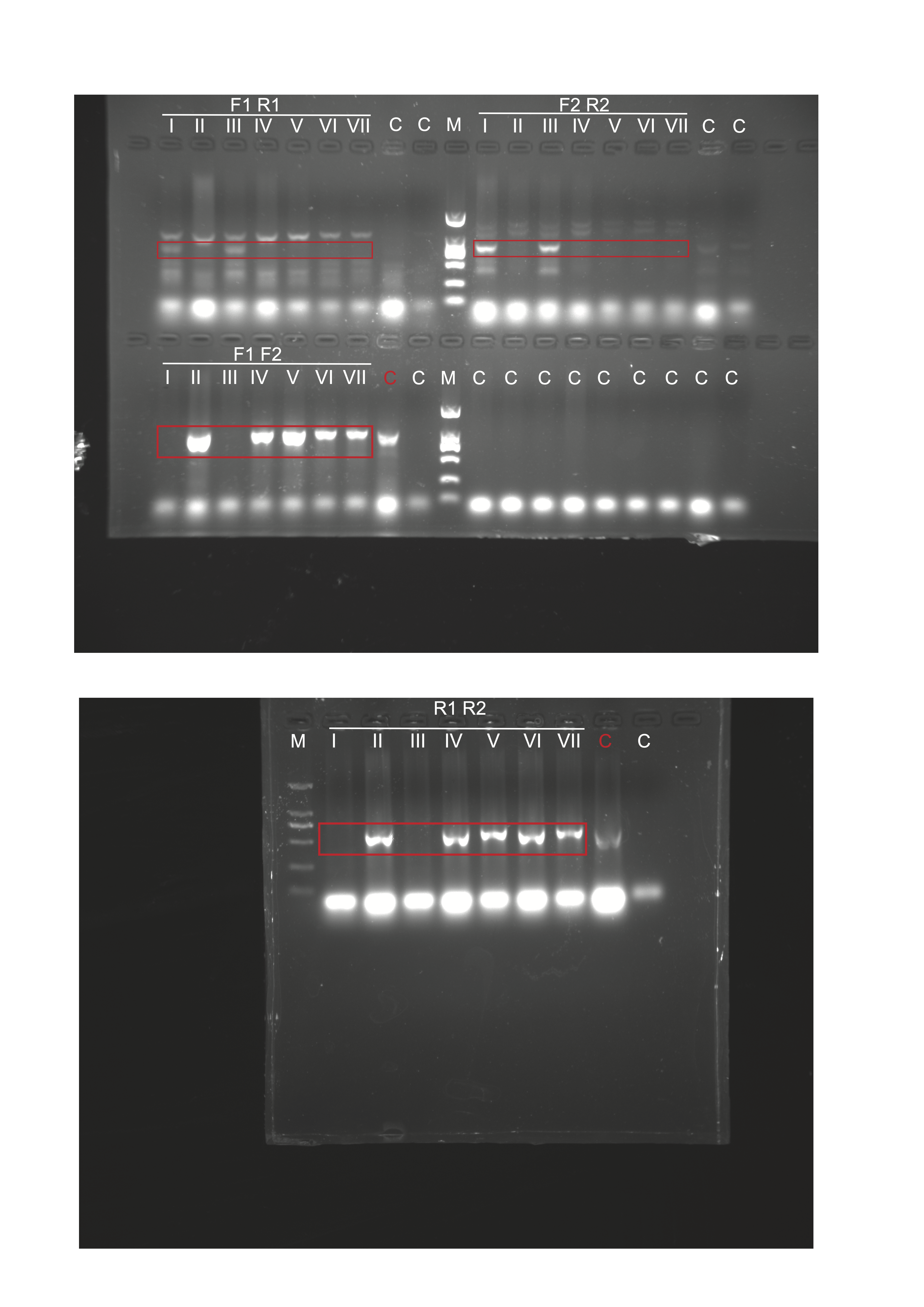

Supplement: Supplementary file 6 — Additional file 6. [file 12864_2021_8212_MOESM6_ESM.png]

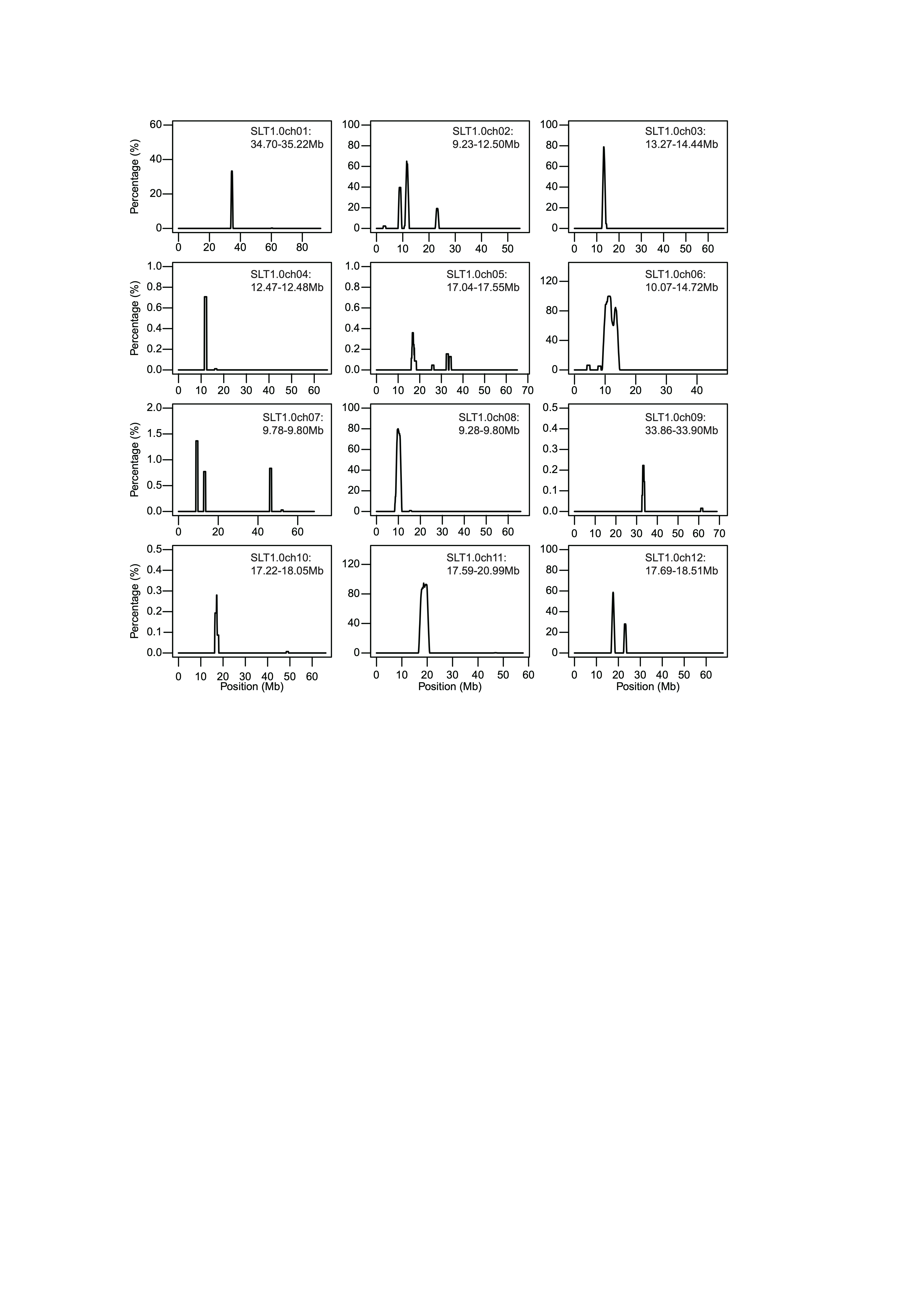

Supplement: Supplementary file 9 — Additional file 9. [file 12864_2021_8212_MOESM9_ESM.png]

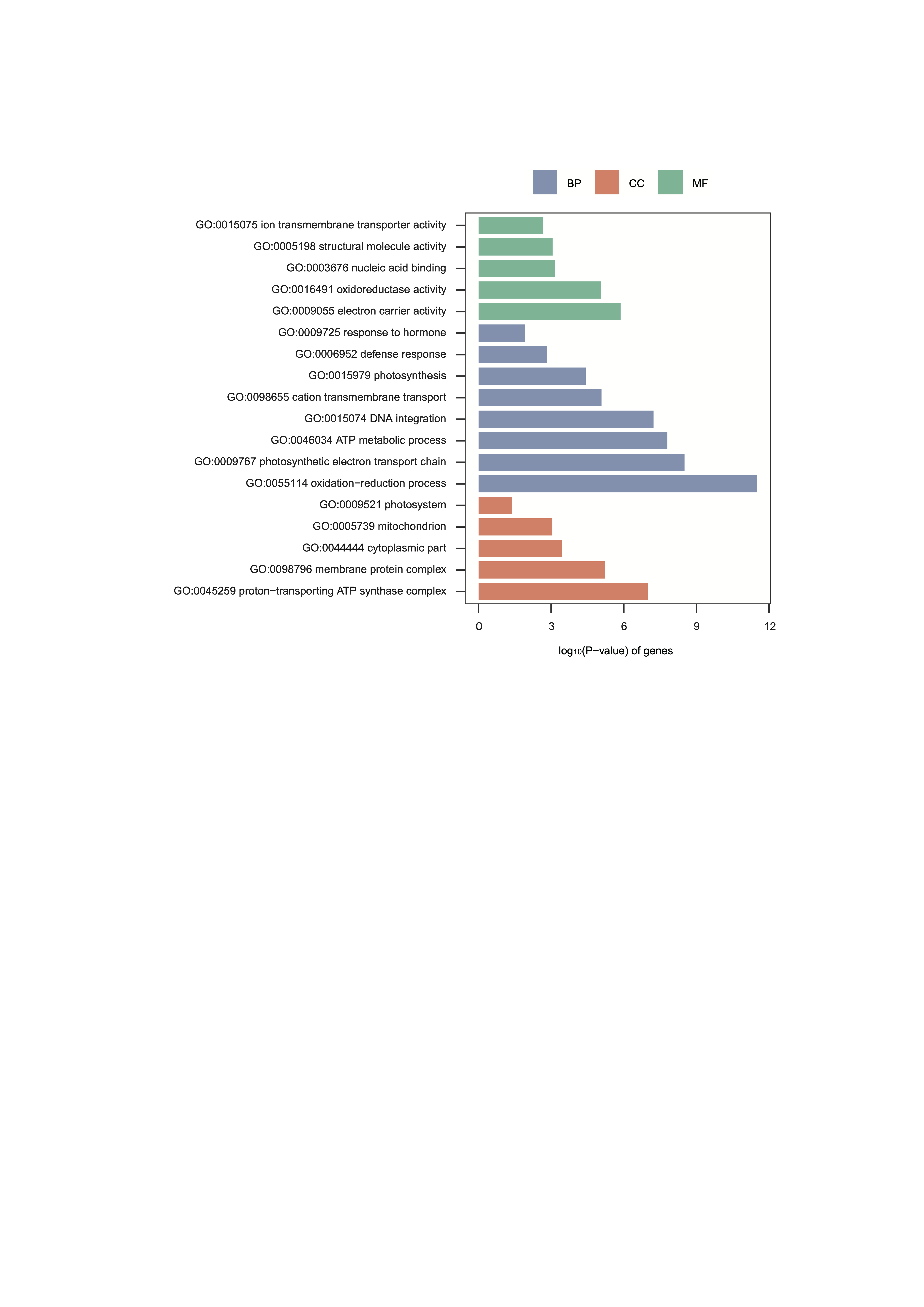

Supplement: Supplementary file 10 — Additional file 10. [file 12864_2021_8212_MOESM10_ESM.png]

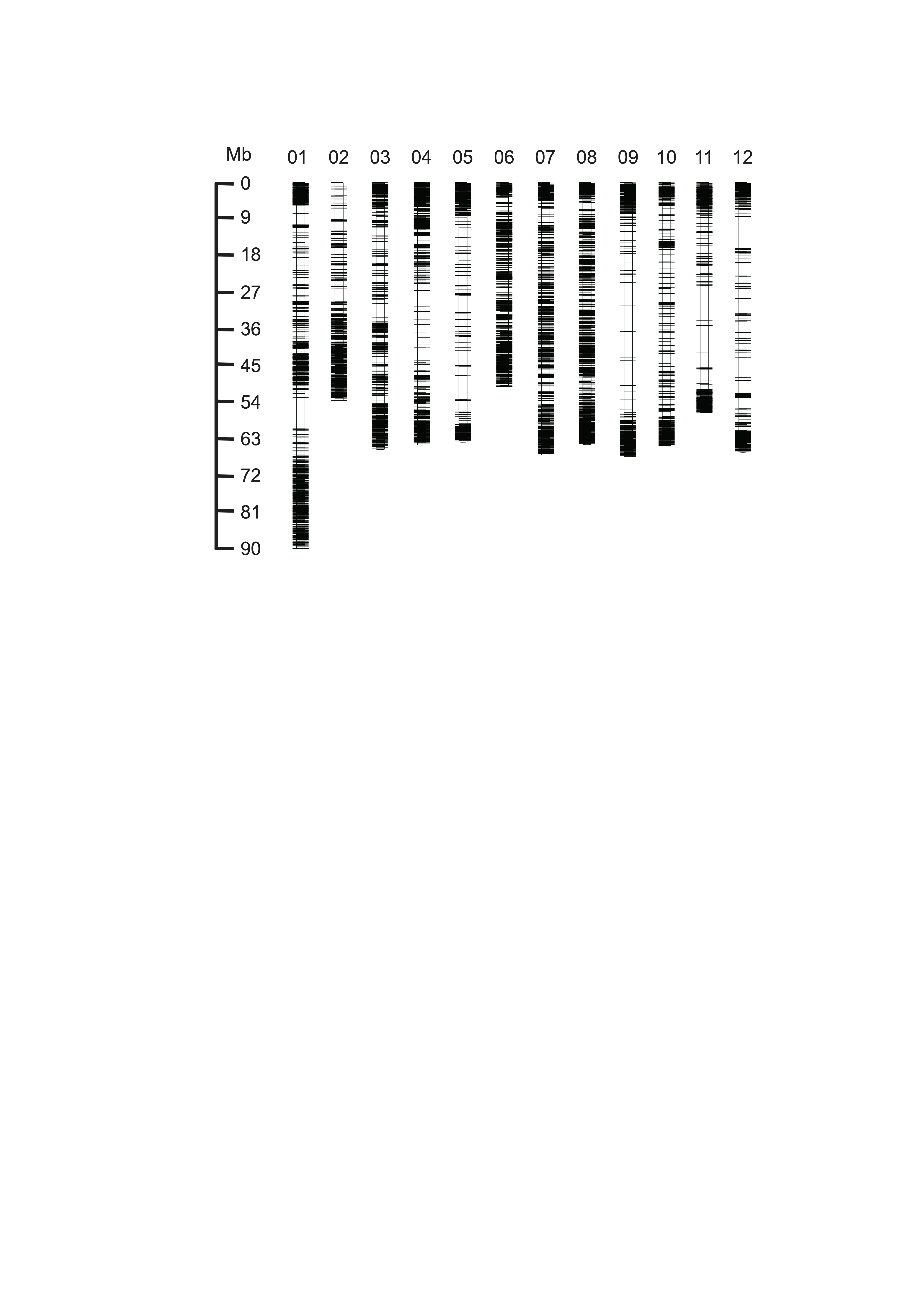

Supplement: Supplementary file 11 — Additional file 11. [file 12864_2021_8212_MOESM11_ESM.png]
